# Mid- to long-term mechanical performance of left bundle branch area pacing: a comprehensive echocardiographic comparison of capture modalities

**DOI:** 10.1093/europace/euaf271

**Published:** 2025-10-29

**Authors:** Álvaro Estévez Paniagua, Sem Briongos-Figuero, Manuel Tapia Martínez, Silvia Jiménez Loeches, Ana Sánchez Hernández, Delia Heredero Palomo, Elena Sánchez López, Arantxa Luna Cabadas, Roberto Muñoz-Aguilera

**Affiliations:** Cardiology Department, Hospital Infanta Leonor Hospital, Gran Vía del Este 80, Madrid 28030, Spain; Cardiology Department, Hospital Infanta Leonor Hospital, Gran Vía del Este 80, Madrid 28030, Spain; Cardiology Department, Hospital Central de la Defensa Gómez Ulla, Glorieta del Ejército, 1, Madrid 28047, Spain; Cardiology Department, Hospital Infanta Leonor Hospital, Gran Vía del Este 80, Madrid 28030, Spain; Cardiology Department, Hospital Infanta Leonor Hospital, Gran Vía del Este 80, Madrid 28030, Spain; Cardiology Department, Hospital Infanta Leonor Hospital, Gran Vía del Este 80, Madrid 28030, Spain; Cardiology Department, Hospital Infanta Leonor Hospital, Gran Vía del Este 80, Madrid 28030, Spain; Cardiology Department, Hospital Infanta Leonor Hospital, Gran Vía del Este 80, Madrid 28030, Spain; Cardiology Department, Hospital Infanta Leonor Hospital, Gran Vía del Este 80, Madrid 28030, Spain

**Keywords:** Left bundle branch area pacing, Left bundle branch pacing, Left ventricular septal pacing, Myocardial work, Echocardiography

## Abstract

**Aims:**

Left bundle branch area pacing (LBBAP) electrical performance is well documented. Nevertheless, the long-term mechanical implications, and differences between left bundle branch pacing (LBBP) and left ventricular septal pacing (LVSP), remain unclear. This study assessed mechanical performance of LBBAP in a mid- to long-term post-implantation setting and compared capture types.

**Methods and results:**

In this prospective, single-centre study, 88 patients with preserved left ventricular ejection fraction and successful LBBAP underwent paired echocardiography during intrinsic and paced rhythm (median 18 months post-implant). Two-dimensional, speckle-tracking and myocardial work (MW) indices were analysed. Adjusted linear mixed-effects models for repeated measures compared LBBAP vs. intrinsic rhythm and LBBP vs. LVSP differences. Independent predictors of changes in MW indices were sought with multivariate regression. Left bundle branch area pacing showed lower left ventricular volumes than intrinsic rhythm and a decrease in global longitudinal strain (16.2% vs. 16.8%, *P* = 0.040). Global constructive work (2232.8 vs. 2028.8 mmHg%, *P* < 0.001) and global wasted work (266.2 vs. 219.1 mmHg%, *P* = 0.003) were higher during pacing, without differences in global work index (1607.8 vs. 1536.7 mmHg%, *P* = 0.171) or global work efficiency (GWE) (88.3% vs. 89.3%, *P* = 0.092). Between LBBP (*n* = 63) and LVSP (*n* = 25), no statistically significant differences were found in any parameter after covariate adjustment (all *P* > 0.28). In multivariate analysis, LVSP independently predicted modest GWE decrease.

**Conclusion:**

In patients with preserved LVEF, LBBAP maintains global mechanical performance comparable to intrinsic rhythm while increasing constructive and wasted work. Mechanical performance is largely similar between LBBP and LVSP, supporting the physiological value of LBBAP regardless of capture type.

## Graphical Abstract

**Graphical Abstract:**
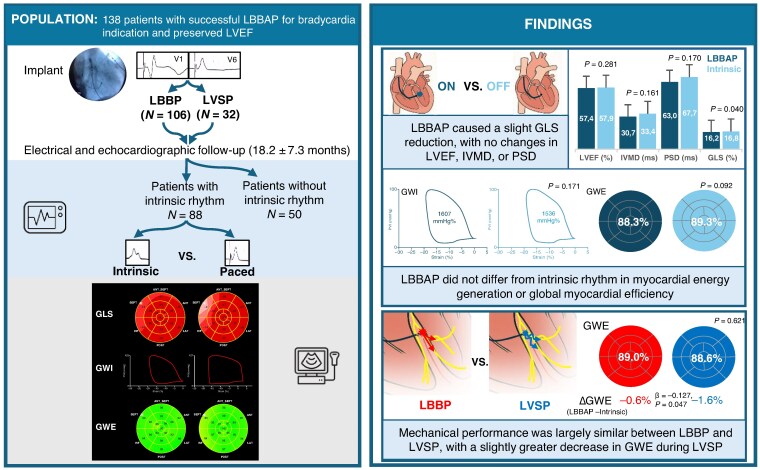
LBBAP, left bundle branch area pacing; LVEF, left ventricular ejection fraction; LBBP, left bundle branch pacing; LVSP, left ventricular septal pacing; IVMD, interventricular mechanical delay; PSD, peak strain dispersion; GLS, global longitudinal strain; GWE, global work efficiency; GWI, global work index.

What’s new?

- Left bundle branch area pacing (LBBAP) preserves global mechanical function compared with intrinsic rhythm while modestly enhancing systolic constructive and wasted work.
- Mechanical performance is largely similar between left bundle branch pacing and left ventricular septal pacing, with a subtle effect on global work efficiency.
- Mechanical performance patterns closely mirror those previously described in the acute phase, reinforcing the consistency of LBBAP over time and its role as a preferred pacing strategy over non-physiological approaches.

## Introduction

Left bundle branch area pacing (LBBAP) is the result of an important evolution in the field.^1^ It is achieved by capturing the left bundle (LB) system at any point [left bundle branch pacing (LBBP)] or by pacing the endocardium within the left side of the interventricular septum [left ventricular septal pacing (LVSP)].^2^ Left bundle branch area pacing modalities intend to replicate a normal left bundle system activation, in order to restore or preserve left ventricular (LV) synchronous contraction, although electrical heterogeneity exists depending on the final position of the lead inside the septum, affecting both LV and right ventricular (RV) activations.^3,4^

Established ECG criteria, defined to differentiate LBBP from LVSP, assume that fast LV activation, provided by the capture of the LBB, leads to LV electrical synchrony, along with delayed RV activation. The mechanical effect of late RV depolarization is uncertain, although it seems that has little importance in acute haemodynamics.^5^ Left ventricular electrical synchrony should be followed by LV mechanical synchronous contraction. There are several reports on the echocardiographic and clinical benefits of LBBAP, both in anti-bradycardia and heart failure indications.^6,7^ However, most of the current studies are focused on electrical synchrony outcomes,^8–11^ but is not clear how and to what extent these electrical parameters relate to mechanical performance.^12–14^ Moreover, little is known about the hypothetical differences between the LBBAP modalities in that benefit.^15–17^ Left ventricular septal pacing has been suggested as a good alternative to LBBP, as it also results in a better LV activation than conventional RV pacing.^18^ So far, differences between LBBP and LVSP in mechanical synchrony assessed by myocardial work (MW) had only been described in virtual models.^19^ More recently, real-life data using MW have also reported mechanical disparities between both capture types.^17^ By contrast, Cano *et al.*,^20^ using conventional echocardiography, found no significant differences between LBBP and LVSP.

Transthoracic echocardiography remains the standard for assessing LV function, with ejection fraction (LVEF) still the most widely used index. However, LVEF has limitations, and subclinical dysfunction may go unnoticed. Global longitudinal strain (GLS) improves sensitivity^21^ but is still afterload dependent. Myocardial work is a novel, non-invasive technique that incorporates LV pressure (from cuff blood pressure) into strain analysis.^22,23^ From pressure–strain loops, several indices can be derived: the global work index (GWI) reflects total MW; the global constructive work (GCW) represents effective energy contributing to ventricular ejection and relaxation; the global wasted work (GWW) quantifies energy lost through ineffective shortening and lengthening; and the global work efficiency (GWE) expresses the balance between GCW and GWW. These indices add value beyond LVEF and GLS in evaluating LV mechanics, providing a less load-dependent measure.

We aimed to investigate the impact of LBBAP on left ventricular mechanical performance in a single-point of a mid- to long-term post-implantation period, assessed through comprehensive echocardiographic evaluation including MW indices. Specifically, our objectives were (i) to compare mechanical function between intrinsic and paced rhythm in patients with preserved LVEF and (2) to assess potential differences between LBBP and LVSP modalities.

## Methods

### Study population

This is a single-centre and prospective cohort study. Consecutive patients with an attempt of LBBAP procedure for bradycardia pacing indication at our institution were screened. Inclusion criteria were as follows:

1. Successful LBBAP procedure
2. Preserved LVEF > 50% at implant
3. Ability to understand the nature of the study and give approved consent

Patients with any degree of left ventricular systolic dysfunction and those with severe left valvular heart disease at implant were excluded for this analysis. Other exclusion criteria are described in the Supplementary material.

The study adhered to the Helsinki Declaration as revised in 2013, and the Institutional Bioethical Committee approved the research protocol. All patients were informed about the nature of the conduction system pacing device and provided informed consent.

### Left bundle branch area pacing implant description

In our laboratory, LBBAP implantation is routinely performed using the ‘single lead technique’.^24,25^ Procedures were performed using Medtronic C315His sheath and 3830-69 lumenless active fixed helix screw-in lead, or Boston Scientific SSCP sheaths and stylet-driven leads. We targeted the area located ∼2 cm apically from the tricuspid annulus to perform RV septal pacemapping before the lead deployment. Initial paced QRS morphologies sought were those with QS and notched nadir in V1 with discordant polarity in Lead II and III (R-wave in Lead II with RS/rS/QS morphology in Lead III). The lead screwing-in process was then initiated. Interrupted pacing from the lead was provided to monitor changes in the paced QRS morphology during the penetration process. Paced QRS morphology was considered optimal if significant QRS narrowing occurred, along with the appearance of QR/Qr or rsR′/rSr′ pattern in Lead V1 and RS or R morphology in Lead V5/V6. At this point, QRS measurements and differential output pacing were performed to confirm LBB capture. If optimal paced QRS morphology was not obtained or deep septal lead penetration was not possible, the lead was extracted, and other areas of the interventricular septum (IVS) were targeted.

All procedures were recorded at 100 mm/s sweep speed on a digital electrophysiological system (General Electric, USA).

### Definition of left bundle branch capture and left ventricular septal capture at implant

Left bundle branch capture was confirmed if unipolar paced QRS morphology in Lead V1 showed QR/rSR′ pattern and at least one of the following:

1. QRS morphology transition:
  - From non-selective (ns)-LBBP to selective (s)-LBBP during threshold test characterized by isoelectric interval before the local EGM and the appearance of M/rsR′ pattern and wide R′ with a notch in Lead V1, S wave in V5/V6, with constant V6-RWPT.^2^
  - From ns-LBBP to LVSP capture defined by an abrupt prolongation of V6-R-wave peak time (V6-RWPT) ≥ 10 ms at decreasing output during threshold test^26^ or during lead screwing-in process.^27^
2. When differential output pacing could not be demonstrated, the following ECG-based criteria were used to confirm LBB capture: V6-RWPT < 75 ms in patients with non-diseased LBB, V6-RWPT < 80 ms in patients with diseased LBB, or V6-V1 interpeak interval > 44 ms.^2^

Left ventricular septal pacing was defined when the paced QRS morphology at deep septal location showed a QR or QS pattern in Lead V1, R-wave without any notch in Lead V6, and none of the above LBBP criteria were met.

### Estimation of left bundle branch capture and left ventricular septal capture at follow-up

As transition criteria were not able to be applied, LBB capture was considered if unipolar paced QRS showed at least one of the following:

1. V6-RWPT < 75 ms in patients with non-diseased LBB or V6-RWPT < 80 ms in patients with diseased LBB
2. V6-V1 interpeak interval > 44 ms^2^
3. When LBBP was demonstrated at implant and V6-RWPT did not differ more than 5 ms at follow-up

Left ventricular septal pacing was considered when the paced QRS morphology showed a QR or QS pattern in Lead V1, R-wave without any notch in Lead V6, and none of the above estimations were met.

### Follow-up visit

Data acquisition was performed in all enrolled patients during the second out-of-hospital follow-up visit, which is currently scheduled ∼18 months after implantation. The study protocol visit consisted of the following:

1. Device check-up and electrocardiogram acquisition

A device interrogation was done, and electrical and programming data were acquired. A manual threshold test was performed in unipolar configuration to confirm the LBBAP lead threshold. A 12-lead ECG was obtained after programming in VVI mode (regardless of the baseline rhythm) with a lower rate at least 10 b.p.m. over patient’s intrinsic heart rate and unipolar pacing configuration to ensure LBBA capture without fusion. Electrocardiographic measurements were performed using Cardiosoft software (GE Healthcare, USA), with digital callipers and sweep speed set to 100–200 mm/s.

2. Echocardiographic acquisition and analysis

All patients underwent an echocardiogram performed by an experienced technician (T.H.), blinded to ECG and pacing parameters, with GE Vivid E95 ultrasound equipment (GE Company, USA). Standard echocardiographic images of the parasternal, apical two-, three-, and four-chamber views, for at least five cardiac cycles, were recorded and stored in DICOM form. Pulse-wave Doppler images of LV and RV outflow tracts were acquired. Non-invasive blood pressure recordings were taken by a brachial artery sphygmomanometer at the same time. Two echocardiographic studies were performed: one during paced rhythm and, if possible, another during spontaneous rhythm. Both studies were acquired with a washout period of at least a 30-min delay. To ensure complete LBBAP in patients with intrinsic rhythm, VVI mode 10 beats/min over patient’s intrinsic heart rate and unipolar pacing configuration were programmed. Otherwise, pacing mode was not modified, and unipolar pacing was confirmed (see Supplementary material for the detailed protocol).

The quantification of MW, mechanical synchrony parameters, and LVEF was conducted by EchoPAC software (GE Healthcare). In the Q-analysis program, two-dimensional images from the standard apical views were selected first. After three index points defining the mitral annulus and ventricular apex, left ventricular endo- and epicardium were tracked automatically, with manual adjustment if necessary. Then, the opening/closing time of mitral and aortic valves were determined according to pulsed-wave Doppler imaging. Pressure–strain loops were constructed automatically by integrating LV strain with blood pressure, and MW parameters were calculated. If regional tracking was suboptimal in more than two segments from one single view, the strain and MW analysis should be avoided. Interventricular mechanical delay (IVMD) was measured by pulsed-wave Doppler as the interval between the pulmonary and aortic pre-ejection time. Peak strain dispersion (PSD) was calculated from the standard deviation of time to peak systolic strain from 17 segments and was used to evaluate intraventricular mechanical synchronization. Global longitudinal strain was calculated by speckle-tracking analysis using standard apical views (long-axis, two-chamber, and four-chamber). Global constructive work (work performed by systolic shortening and myocardial lengthening in the isovolumetric relaxation phase),^22^ GWW (work performed by systolic lengthening and myocardial shortening in the isovolumetric relaxation phase), GWE (the ratio between constructive work and the sum of wasted and constructive work), and GWI (work performed during the period from mitral valve closure to mitral valve opening) were acquired.

### Measurements of electrocardiographic parameters

At least three QRS complexes were used for all measurements, both at implant and follow-up, and the values were averaged. In each patient, every available paced QRS type (LBBP and LVSP) and native QRS was measured. The following QRS characteristics were obtained:

1. Native QRS duration and paced QRS duration measured from the earlier onset and from the pacing stimulus to the latest offset of the QRS in any of the 12 ECG leads recorded simultaneously.
2. R-wave peak time, measured from the beginning of the pacing spike to the peak of R-wave in leads V6 and V1.
3. V6-V1 interpeak interval, measured from the R-wave peak in lead V6 to the R-wave peak in lead V1.
4. Left bundle branch disease was considered whenever ECG showed either LBB block, bifascicular block, or isolated left fascicular block with QRS ≥ 110 ms.

### Statistical analysis

Normality of continuous variables was assessed with the Shapiro–Wilk test. Continuous data are presented as mean ± SD or median [IQR] and categorical data as counts and percentages. Group comparisons used the Student’s *t*-test or Mann–Whitney *U* test for continuous variables and χ² or Fisher’s exact test for categorical variables.

Inter-observer reproducibility of electrical and echocardiographic measurements was evaluated with the intra-class correlation coefficient and Bland–Altman analysis (see Supplementary material).

To explore potential differences in echocardiographic parameters between intrinsic rhythm and LBBAP, as well as between LBBP and LVSP, we applied a two-step approach. First, unadjusted comparisons were performed using the paired Student’s *t*-test or Wilcoxon signed-rank test for within-patient rhythm changes (LBBAP vs. intrinsic) and the independent Student’s *t*-test or Mann–Whitney *U* test for between-group pacing modality differences (LBBP vs. LVSP). Second, adjusted analyses were carried out with linear mixed-effects models (LMMs) including random intercepts at the patient level to account for repeated measures. These models tested the main effects of rhythm and pacing modality, as well as their interaction, adjusted for age, sex, and baseline LVEF. Separate models were run for conventional 2D parameters, strain-derived indices, and MW components. Pairwise comparisons were Bonferroni adjusted.

Finally, univariate and multivariate linear regression analyses were used to identify predictors of MW changes (ΔMW, calculated as paced minus intrinsic values). Variables with *P* < 0.10 in univariate analysis were entered into multivariate models. Multivariate regressions were adjusted for age, sex, baseline LVEF and GLS, % of ventricular pacing, stimulated QRS duration, and changes in other MW indices where appropriate. A *P* value of <0.05 was considered statistically significant. All statistical analyses were performed using SPSS, version 20.0 (IBM Corporation, Chicago, Illinois).

## Results

### Study population

**Figure 1:**
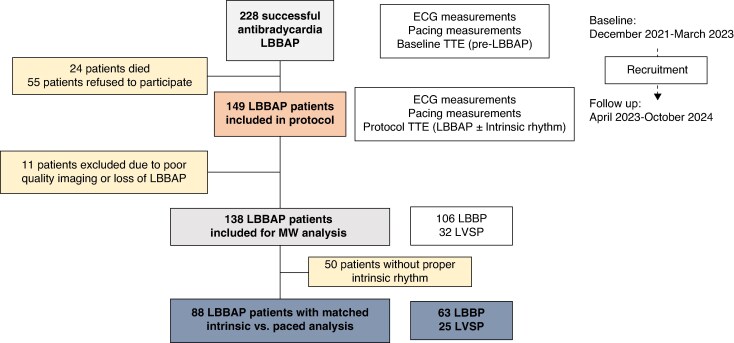
Study population enrolment flowchart. TTE, transthoracic echocardiogram; MW, myocardial work; LBBAP, left bundle branch area pacing; LBBP, left bundle branch pacing; LVSP, left ventricular septal pacing.

A total of 228 patients undergoing successful LBBAP implant, who met all the inclusion criteria and none of the exclusion criteria, were screened. Twenty-four patients died (see Supplementary material), and 55 patients refused to participate. A total of 149 patients who fulfilled the study protocol were studied. Of these 149 patients, 11 patients were excluded due to inadequate image quality (*n* = 10) or loss of LBBAP (*n* = 1). Among the remaining 138 patients who underwent echocardiographic protocol, paired measurements (intrinsic rhythm vs. rhythm with LBBAP) were obtained in 88 patients (*Figure 1*). Follow-up visit was performed 18.2 ± 7.3 months after the implant. Baseline characteristics of the study population are depicted in *Table 1*. Sixty-three patients showed LBBP and 25 patients LVSP.

**Table 1:** General characteristics of the study population and stratification according to pacing modality

	All patients (*n* = 88)	LBBP (*n* = 63)	LVSP (*n* = 25)	*P* value
Age (years)	78.9 ± 8.4	78.0 ± 9.0	79.7 ± 7.0	0.293
Female	43 (48.9%)	33 (52.4%)	10 (40.0%)	0.349
Hypertension	79 (89.8%)	55 (87.3%)	24 (96.0%)	0.436
Diabetes	29 (33.0%)	21 (33.3%)	8 (32.0%)	1.000
Atrial fibrillation	37 (42.0%)	29 (46.0%)	8 (32.0%)	0.338
Heart failure	10 (11.4%)	9 (14.3%)	1 (4.0%)	0.270
Coronary heart disease	11 (12.5%)	9 (14.3%)	2 (8.0%)	0.722
COPD	6 (6.8%)	5 (7.9%)	1 (4.0%)	0.419
CKD^a^	13 (14.8%)	10 (15.9%)	3 (12.0%)	0.751
Medical treatment				
Anticoagulant	41 (47.1%)	29 (46.8%)	12 (48.0%)	1.000
Beta-blocker	25 (28.4%)	19 (30.2%)	6 (24.0%)	0.612
ACEI/ARA-II/ARNI	58 (65.9%)	41 (65.0%)	17 (68.0%)	0.806
MRA	8 (9.4%)	7 (11.5%)	1 (4.2%)	0.431
Pacing indication				
AVB/BF block/alternant BBB	34 (38.6%)	24 (38.1%)	10 (40.0%)	1.000
Slow AF/SND	54 (61.4%)	39 (61.9%)	15 (60.0%)	
Intrinsic QRS (at follow-up) (milliseconds)	115.5 ± 28.2	115.3 ± 28.7	117.0 ± 28.2	0.557
Intrinsic wide QRS (≥120 ms)	38 (43.2%)	28 (44.4%)	10 (40.0%)	0.813
Diseased LBB (≥110 ms)	24 (27.3%)	16 (25.4%)	8 (32.0%)	0.599
Paced QRS (at follow-up) (milliseconds)				
From stimulus	138.0 (131.0–143.0)	134.0 (129.0–141.0)	141.0 (136.0–160.0)	0.021^b^
From onset	108.0 (101.0–115.7)	108.0 (98.0–115.0)	111.0 (108.0–125.5)	0.010^b^
Device type				
SR	15 (17.0%)	14 (22.0%)	1 (4.0%)	0.057
DR	73 (83.0%)	49 (77.8%)	24 (96.0%)	
Intrinsic LVEF (%)	57.8 ± 5.1	57.8 ± 5.4	57.8 ± 4.5	1.000
LVEDD (mm)	44.7 ± 5.4	44.4 ± 5.5	45.8 ± 5.2	0.402
Interventricular septum thickness (mm)	12.0 ± 2.7	12.0 ± 2.7	12.9 ± 1.9	0.151
Left atrial volume (mL/m^2^)	38.0 (31.0–50.0)	38.0 (30.5–52.2)	38.0 (31.5–49.7)	0.971^b^
Ventricular pacing at follow-up (%)	3.3 (0.1–42.9)	4.2 (0.4–52.9)	3.3 (0.1–42.9)	0.457^b^

Values are mean ± standard deviation (SD), median (interquartile range), and *n* (%).

LBBP, left bundle branch pacing; LVSP, left ventricular septal pacing; COPD, chronic obstructive pulmonary disease; CKD, chronic kidney disease; ACEI, angiotensin-converting enzyme inhibitor; ARA-II, angiotensin II receptor antagonist; ARNI, angiotensin receptor–neprilysin inhibitor; AVB, atrioventricular block; BF block, bifascicular block; BBB, bundle branch block; LVEF, left ventricular ejection fraction; LVEDD, left ventricular end-diastolic diameter.

^a^Glomerular filtration rate < 60 mL/min/1.73 m^2^.

^b^Non-parametric tests.

Comparison between patients with and without intrinsic rhythm at follow-up is provided in Supplementary material online, *Table S1*.

### Left bundle branch area pacing electrical parameters at follow-up

Left bundle branch area pacing demonstrated stability during follow-up; only one patient, who initially had LVSP capture at implantation, lost capture over time. As we have previously reported in this cohort, V6-RWPT, V1-RWPT, interpeak interval, and QRS duration decreased significantly from baseline (see Supplementary material online, *Table S2*).^28^ Differences according to LBBAP modality (LBBP vs. LVSP) are presented in Supplementary material online, *Table S2*.

### Intrinsic rhythm vs. left bundle branch area pacing

- 2D-echocardiographic parameters

Paired analysis revealed no significant changes in LVEF (58.0 [54.0–62.0] % vs. 57.0 [55.0–60.0] %, *P* = 0.247) or IVMD (30.0 [20.0–43.0] ms vs. 26.0 [18.0–40.0] ms, *P* = 0.079) between intrinsic rhythm and LBBAP (*Table 2*). These results were confirmed in the adjusted model (LVEF: *F* = 1.177, *P* = 0.281; IVMD: *F* = 1.994, *P* = 0.161), indicating negligible effects. Left ventricular end-diastolic volume (LVEDV) and left ventricular end-systolic volume (LVESV) were both significantly reduced during pacing in the unadjusted analysis (LVEDV: 72.0 [59.0–90.0] ml vs. 70.0 [55.0–85.0] ml, *P* = 0.008; LVESV: 33.6 [25.0–41.7] ml vs. 31.0 [25.0–40.0] ml, *P* = 0.009), and these differences were corroborated after adjusting for age, sex, and LVEF (LVEDV: *F* = 8.611, *P* = 0.004; LVESV: *F* = 6.330, *P* = 0.014) (*Table 2*).

- Myocardial strain and MW

**Table 2:** Unadjusted and adjusted 2D-echocardiographic, strain, and MW index comparisons between LBBAP and intrinsic rhythm

	Unadjusted model *t-*test/Wilcoxon test			Adjusted model LMM within subject analysis (LBBAP vs. Intrinsic)				
	LBBAP (*n* = 88)	Intrinsic (*n* = 88)	*P* value	LBBAP (*n* = 88)	Intrinsic (*n* = 88)	*F* (df)	*P* value	Pairwise comparison (Bonferroni)
LVEF (%)	57.0 (55.0–60.0)	58.0 (54.0–62.0)	0.247^a^	57.4 (56.4–58.4)	57.9 (56.9–59.0)	1.177 (95.2)	0.281	LBBAP = Intrinsic (ns)
LVEDV (mL)	70.0 (55.0–85.0)	72.0 (59.0–90.0)	0.008^a^	72.8 (69.1–76.5)	76.6 (72.6–80.5)	8.611/85.0)	0.004	LBBAP < Intrinsic
LVESV (mL)	31.0 (25.0–40.0)	33.6 (25.0–41.7)	0.009	34.1 (32.0–36.2)	36.3 (34.0–38.5)	6.330 (85.1)	0.014	LBBAP < Intrinsic
IVMD (ms)	26.0 (18.0–40.0)	30.0 (20.0–43.0)	0.079^a^	30.7 (27.5–33.9)	33.4 (29.8–37.1)	1.994 (95.7)	0.161	LBBAP = Intrinsic (ns)
PSD (ms)	59.0 (50.0–70.0)	65.0 (50.7–77.0)	0.013^a^	63.0 (58.1–67.8)	67.7 (62.0–73.4)	1.913 (105.2)	0.170	LBBAP = Intrinsic (ns)
GLS (%)	−15.6 ± 2.5	−16.4 ± 2.6	0.002	16.2 (15.6–16.7)	16.8 (16.1–17.4)	4.375 (78.9)	0.040	LBBAP < Intrinsic
GWI (mmHg%)	1525.8 ± 378.8	1504.6 ± 388.9	0.652	1607.8 (1523.5–1692.1)	1536.7 (1439.3–1634.1)	1.909 (85.5)	0.171	LBBAP = Intrinsic (ns)
GCW (mmHg%)	2084.5 (1834.7–2426.2)	1973.0 (1730.5–2173.0)	0.001^a^	2232.8 (2143.8–2321.9)	2028.8 (1928.8–2128.1)	20.424 (78.8)	<0.001	LBBAP > Intrinsic
GWW (mmHg%)	249.5 (173.0–363.2)	177.0 (110.7–323.2)	0.001^a^	266.2 (238.4–294.0)	219.1 (187.5–250.7)	9.293 (84.8)	0.003	LBBAP > Intrinsic
GWE (%)	88.0 (85.0–92.0)	90.0 (85.0–93.0)	0.044	88.3 (87.3–89.3)	89.3 (88.2–90.4)	2.905 (91.1)	0.092	LBBAP = Intrinsic (ns)

Unadjusted values are mean ± standard deviation (SD) or median (interquartile range). Adjusted values are estimated marginal mean (95% CI).

LBBAP, left bundle branch area pacing; df, degrees of freedom; LVEF, left ventricular ejection fraction; LVEDV, left ventricular end-diastolic volume; LVESV, left ventricular end-systolic volume; IVMD, interventricular mechanical delay; PSD, peak strain dispersion; GLS, global longitudinal strain; GWI, global work index; GCW, global constructive work; GWW, global wasted work; GWE, global work efficiency.

^a^Non-parametric tests.

Paired analysis showed significant reductions in GLS (−16.4 ± 2.6% vs. −15.6 ± 2.5%, *P* = 0.002) and PSD (65.0 [50.7–77.0] ms vs. 59.0 [50.0–70.0] ms, *P* = 0.013) during LBBAP, but only the difference in GLS remained significant after adjustment (GLS: *F* = 4.375, *P* = 0.040; PSD: *F* = 1.913, *P* = 0.170) (*Table 2*).

**Figure 2:**
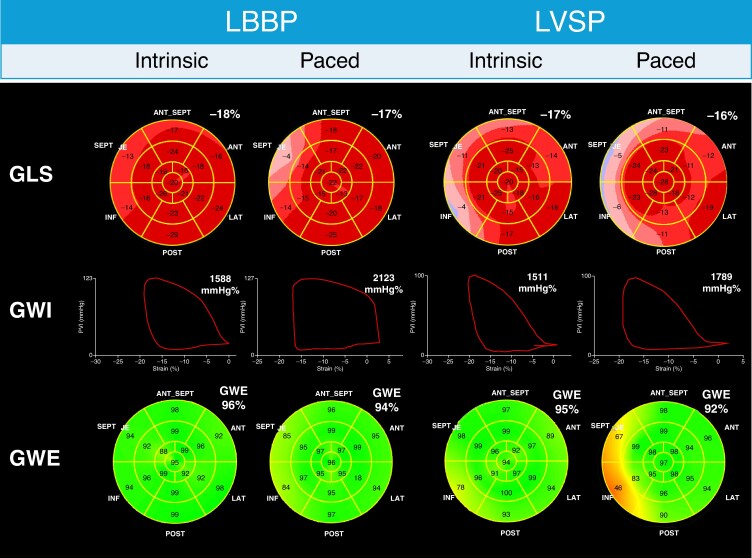
Intra-patient and inter-group comparisons of the main strain and MW indices. The figure shows representative examples from two patients enrolled in the study. These cases illustrate the overall trends observed in our cohort: modest decrease in GLS and neglect difference in GWE during LBBAP. Also, local disparities may be appreciated in GLS and GWE, with worse septal performance during LBBAP. LBBP, left bundle branch pacing; LVSP, left ventricular septal pacing; GLS, global longitudinal strain; GWI, global work index; GWE, global work efficiency.

For MW indices, the unadjusted analysis showed increases in GCW (1973.0 [1730.5–2173.0] vs. 2084.5 [1834.7–2426.2] mmHg%, *P* = 0.001) and GWW (177.0 [110.7–323.2] vs. 249.5 [173.0–363.2] mmHg%, *P* = 0.001) during pacing compared with intrinsic rhythm, with no significant differences in GWI (*P* = 0.652). Global work efficiency was significantly lower during pacing in the unadjusted analysis (90.0 [85.0–93.0] % vs. 88.0 [85.0–92.0] %, *P* = 0.044). After adjustment, GCW (*F* = 20.424; *P* < 0.001) and GWW (*F* = 9.293; *P* = 0.003) were significantly higher with pacing, while GWI remained non-significant (*F* = 1.909; *P* = 0.171), and GWE was no longer significant (*F* = 2.905; *P* = 0.092) (*Figure 2*), suggesting that the small apparent reduction in efficiency was not independent of covariate effects (*Table 2*). Noteworthy, during LBBAP, in the unadjusted analysis, no significant differences were observed in GWE, GCW, or GWW, between patients with and without spontaneous rhythm at follow-up. By contrast, significant differences were found for GLS and GWI (see Supplementary material online, *Table S3*).

### Left bundle branch pacing vs. left ventricular septal pacing

- 2D-echocardiographic parameters

There were no significant differences between the two groups, except for paced QRS duration, which was significantly longer with LVSP (*Table 1*). In the unadjusted analysis, no significant differences were found between LBBP and LVSP groups for LVEF, LVEDV, or IVMD in either intrinsic rhythm or pacing condition (all *P* > 0.05), except for LVESV, which was significantly higher in LVSP during intrinsic rhythm (38.8 ± 13.0 mL vs. 32.4 ± 9.9 mL, *P* = 0.034), although this difference disappeared during pacing (34.1 ± 11.5 ml vs. 32.0 ± 10.1 ml, *P* = 0.392) (*Table 3*).

**Table 3:** Unadjusted 2D-echocardiographic, strain, and MW index comparisons between LBBP and LVSP

	Descriptive between subject analysis (*t*-test/*U* Mann–Whitney)			
	Rhythm	LBBP	LVSP	*P* value
LVEF (%)	Intrinsic	58.0 (53.0–62.0)	59.0 (54.0–61.0)	0.973
	Paced	57.0 (55.0–61.0)	57.0 (55.0–59.5)	0.944^a^
LVEDV (mL)	Intrinsic	69.0 (56.0–90.0)	81.0 (63.0–93.0)	0.111
	Paced	67.0 (55.0–85.0)	70.0 (57.5–90.0)	0.944^a^
LVESV (mL)	Intrinsic	33.0 (24.0–39.0)	38.0 (28.0–48.5)	0.034
	Paced	31.0 (24.0–37.0)	33.0 (27.5–40.0)	0.893^a^
IVMD (ms)	Intrinsic	30.0 (20.0–40.0)	30.0 (19.0–47.5)	0.853
	Paced	28.0 (16.2–40.0)	21.0 (19.5–41.5)	0.943^a^
PSD (ms)	Intrinsic	64.5 (50.5–77.0)	65.0 (50.5–78.9)	0.974^a^
	Paced	58.0 (49.2–69.7)	63.5 (52.2–75.0)	0.887^a^
GLS (%)	Intrinsic	−16.4 ± 2.6	−16.3 ± 2.5	0.843
	Paced	−15.5 ± 2.6	−15.6 ± 1.9	0.895
GWI (mmHg%)	Intrinsic	1498.5 ± 377.7	1519.9 ± 425.0	0.829
	Paced	1500.3 ± 383.1	1591.3 ± 381.2	0.339
GCW (mmHg%)	Intrinsic	1948.6 ± 343.9	2088.2 ± 471.8	0.152
	Paced	2070.0 ± 372.1	2337.8 ± 494.1	0.010
GWW (mmHg%)	Intrinsic	162.0 (110.2–321.0)	204.5 (113.7–353.0)	0.450^a^
	Paced	228.5 (167.5–336.2)	306.5 (183.5–381.0)	0.393^a^
GWE (%)	Intrinsic	90.0 (85.0–93.0)	88.5 (85.0–92.5)	0.837
	Paced	89.5 (85.0–92.0)	86.5 (85.0–89.2)	0.120^a^

Values are mean ± standard deviation (SD) or median (interquartile range).

LBBP, left bundle branch pacing; LVSP, left ventricular septal pacing; LVEF, left ventricular ejection fraction; LVEDV, left ventricular end-diastolic volume; LVESV, left ventricular end-systolic volume; IVMD, interventricular mechanical delay; PSD, peak strain dispersion; GLS, global longitudinal strain; GWI, global work index; GCW, global constructive work; GWW, global wasted work; GWE, global work efficiency.

^a^Non-parametric tests.

The adjusted LMM confirmed the absence of a significant main effect of pacing modality on LVEF (*F* = 0.413; *P* = 0.512) and LVEDV (*F* = 0.395; *P* = 0.531) or IVMD (*F* < 0.001; *P* = 0.987) (*Table 4*). For LVESV, a trend towards higher values in LVSP compared to LBBP was observed (*F* = 3.418; *P* = 0.067). Also, a significant rhythm × modality interaction was detected for LVESV (*F* = 6.279; *P* = 0.014), suggesting that differences between pacing modalities for LVESV varied slightly between intrinsic and paced conditions (see Supplementary material online, *Table S4*). No other significant interactions between rhythm and pacing modality were detected for the rest of parameters (all *P* > 0.05).

**Table 4:** Adjusted 2D-echocardiographic, strain, and MW index pairwise comparisons between LBBP and LVSP

	LBBP	LVSP	F (df)	*P* value (global)	Pairwise comparison (Bonferroni)
LVEF (%)	58.0 (57.1–58.9)	57.4 (55.7–59.0)	0.433 (129.5)	0.512	LBBP = LVSP (ns)
LVEDV (mL)	73.5 (69.9–77.1)	75.9 (69.5–82.2)	0.395 (125.5)	0.531	LBBP = LVSP (ns)
LVESV (mL)	33.3 (31.3–35.3)	37.1 (33.5–40.6)	3.418 (123.0)	0.067	LBBP = LVSP (ns)
IVMD (ms)	32.1 (29.2–35.0)	32.0 (27.0–37.0)	<0.001 (121.5)	0.987	LBBP = LVSP (ns)
PSD (ms)	66.3 (62.2–70.5)	64.4 (57.3–71.4)	0.219 (116.5)	0.641	LBBP = LVSP (ns)
GLS (%)	16.7 (16.2–17.2)	16.2 (15.3–17.2)	0.630 (114.3)	0.429	LBBP = LVSP (ns)
GWI (mmHg%)	1576.8 (1500.7–1653.0)	1567.7 (1435.0–1700.3)	0.014 (111.0)	0.906	LBBP = LVSP (ns)
GCW (mmHg%)	2088.7 (2005.9–2171.4)	2172.6 (2026.1–2319.1)	0.965 (113.2)	0.328	LBBP = LVSP (ns)
GWW (mmHg%)	228.4 (202.8–254.0)	256.9 (212.0–301.9)	1.180 (115.5)	0.280	LBBP = LVSP (ns)
GWE (%)	89.0 (88.1–89.9)	88.6 (87.0–90.1)	0.246 (119.1)	0.621	LBBP = LVSP (ns)

Values are estimated marginal mean (95% CI). LBBP, left bundle branch pacing; LVSP, left ventricular septal pacing; df, degrees of freedom; LVEF, left ventricular ejection fraction; LVEDV, left ventricular end-diastolic volume; LVESV, left ventricular end-systolic volume; IVMD, interventricular mechanical delay; PSD, peak strain dispersion; GLS, global longitudinal strain; GWI, global work index; GCW, global constructive work; GWW, global wasted work; GWE, global work efficiency.

### Myocardial strain and myocardial work

Unadjusted between-group comparisons revealed no significant differences in GLS or PSD under either condition (intrinsic or paced). Similarly, GWI, GWW, and GWE showed no significant differences between LBBP and LVSP in either rhythm (all *P* > 0.05) (*Table 3*). In contrast, GCW was significantly higher in LVSP compared to LBBP during pacing (2337.8 ± 494.1 mmHg% vs. 2070.0 ± 372.1 mmHg%, *P* = 0.010), while no difference was found in the intrinsic rhythm condition.

In the adjusted test, the effect of pacing on GLS, PSD, GWI, GWW, and GWE remained non-significant (all *P* > 0.28), while there was no longer significant difference in GCW (*F* = 0.965; *P* = 0.328). Moreover, no significant rhythm × modality interaction was detected for any MW or strain parameter (all *P* >0.18) (see Supplementary material online, *Table S4*).

### Pacing and changes in myocardial work indices: independent predictors

**Figure 3:**
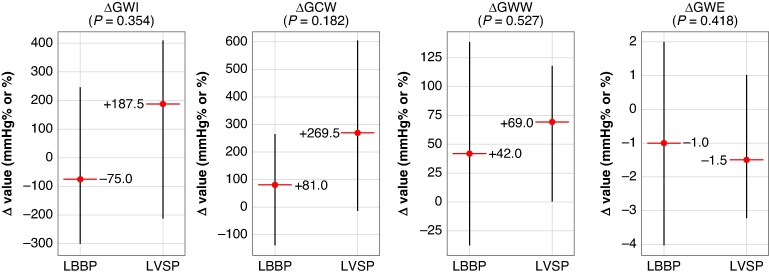
Observed changes in MW indices between paced and intrinsic rhythm. Box-plot graphics showing the changes in MW between LBBP and LVSP groups. Medians values are superimposed.

In multivariate analysis, the change in GWI (ΔGWI) with LBBAP was independently determined only by ΔGCW (β = 0.533; *P* < 0.001). For ΔGCW, the strongest predictors were ΔGWI (β = 0.446; *P* < 0.001), ΔGWW (β = 0.512; *P* = 0.005), and ΔGWE (β = 0.422; *P* = 0.019). The model for ΔGWW demonstrated excellent explanatory power (adjusted *R*² = 0.817), driven primarily by ΔGWE (β = −0.872; *P* < 0.001) and, to a lesser extent, by ΔGCW (β = 0.236; *P* = 0.005) and age (β = 0.159; *P* = 0.024). Finally, the determinants of ΔGWE included ΔGWW (β = −0.891; *P* < 0.001), ΔGCW (β = 0.197; *P* = 0.021), and pacing modality (β = −0.127; *P* = 0.047), with this model achieving a high explanatory capacity (adjusted *R*² = 0.781) (*Table 5*). *Figure 3* illustrates the distribution of ΔGWI, ΔGCW, ΔGWW, and ΔGWE values between LBBP and LVSP, showing the lack of significant inter-group differences in the unadjusted analysis (see Supplementary material online, *Table S5*).

**Table 5:** Multivariate linear regression models for changes in MW indices with LBBAP

	Univariate analysis			Multivariate analysis		
Dependent variable	Independent variable	Standardized β	*P* value	Independent variable	Standardized β	*P* value
ΔGWI	Age	0.351	0.002	ΔGCW	0.533	<0.001
	Intrinsic GLS	0.286	0.012			
	ΔGCW	0.666	<0.001			
	ΔGWE	0.252	0.027			
				*Model adjusted R²*	*0.464*	
ΔGCW	Age	0.345	0.002	ΔGWI	0.446	<0.001
	Diabetes	−0.220	0.054	ΔGWW	0.512	0.005
	Pacing modality	0.215	0.060	ΔGWE	0.422	0.019
	ΔGWI	0.666	<0.001			
	ΔGWW	0.220	0.055			
				*Model adjusted R²*	*0.558*	
ΔGWW	Diabetes	−0.296	0.009	ΔGWE	−0.872	<0.001
	ΔGCW	0.220	0.055	ΔGCW	0.236	0.005
	ΔGWE	−0.825	<0.001	Age	0.159	0.024
				*Model adjusted R²*	*0.817*	
ΔGWE	Diabetes	0.210	0.067	ΔGWW	−0.891	<0.001
	Paced QRS duration^a^	−0.225	0.049	ΔGCW	0.197	0.021
	Paced QRS duration^b^	−0.255	0.025	Pacing modality	−0.127	0.047
	Intrinsic GLS	0.222	0.054			
	ΔGWI	0.252	0.027			
	ΔGWW	−0.825	<0.001			
				*Model adjusted R²*	*0.781*	

Only *P* values < 0.10 in the univariate analysis, and statistically significant predictors in the multivariate analysis, are shown.

GLS, global longitudinal strain; GWI, global work index; GCW, global constructive work; GWW, global wasted work; GWE, global work efficiency.

^a^From stimulus.

^b^From onset.

### Agreement

Inter-observer variability for electrical and echocardiographic parameters showed high agreement for all variables (see Supplementary material online, *Table S6* and *Figure S2*).

## Discussion

In this study, we evaluated both intra-subject differences between LBBAP and intrinsic rhythm and inter-group differences between LBBP and LVSP in a population with preserved LVEF and advanced conduction disease requiring pacing. By integrating conventional 2D echocardiographic parameters, longitudinal strain, and MW indices, we aimed to better delineate the mechanical performance of LBBAP in a stable post-implantation setting.

Overall, in the adjusted comparison, LBBAP was associated with lower LVEDV and LVESV compared with intrinsic rhythm, without changes in LVEF but with a subtle reduction in GLS. The finding of smaller LV volumes during LBBAP may have several explanations. First, patients presented slightly lower heart rates during intrinsic rhythm, resulting in longer diastolic filling times and consequently larger LVEDV. Second, in patients with sinus rhythm and preserved atrioventricular conduction, intrinsic rhythm allows a more physiological contribution of atrial contraction to ventricular filling, which may further enlarge diastolic volumes. A noteworthy finding was the rhythm × capture-type interaction for LVESV, whereby LBBAP pacing led to a greater reduction in LVESV among patients with LVSP compared to those with LBBP. This observation may in part reflect the fact that patients with LVSP exhibited higher baseline LVESV during intrinsic rhythm, making a larger absolute reduction more likely once pacing was established. In any case, these results should be interpreted cautiously, as it cannot be excluded that volumetric parameters may offer complementary insights to strain and MW indices when analysing the pacing effect and comparing its modalities.

In terms of MW, LBBAP was associated with higher GCW and GWW, while GWI and GWE remained unchanged. This pattern indicates that pacing enhances the total amount of constructive systolic work, but also increases wasted work, resulting in no net improvement in efficiency. Interestingly, the combination of increased GCW without significant changes in GWE mirrors findings from other conduction system pacing studies, where higher recruitment was not always accompanied by proportional gains in efficiency.^29^ This could be explained by the persistence of mild residual dyssynchrony despite conduction system capture: anatomical variability in the Purkinje network, the presence of myocardial fibrosis, and electro-mechanical uncoupling may all contribute to regional mechanical delays that are not corrected by restoring fast conduction alone.^30^ This hypothesis is further supported by *in silico* modelling by Meiburg *et al.*,^19^ which demonstrated that even with LBBP, subtle regional dyssynchrony can persist. Notably, although GLS was slightly worse during LBBAP compared with intrinsic rhythm, the absence of significant differences in GWI and GWE supports the added value of MW analysis, as these indices are less load dependent and provide a complementary assessment of myocardial performance.

In the comparison between LBBP and LVSP, no statistically significant differences were found in 2D-echocardiographic, strain, or MW parameters after covariate adjustment and Bonferroni correction. However, in the multivariate analysis, LVSP was a modest independent predictor of lower GWE, even after accounting for the strong influence of GCW and GWW. Although expected, the relationship between GCW, GWW, and GWE reinforces the internal consistence of our results. By contrast, exploring the interplay among GWI, GCW, and GWW may provide complementary information to better characterize the mechanical effects of LBBAP.

Nevertheless, overall mechanical performance appears largely similar between LBBP and LVSP, consistent with the findings by Bertini *et al.*^17,31^ These results appear to align with the detailed electrophysiological study by Rijks *et al.*,^32^ which showed no significant differences in total LV activation time or other advanced electrical dyssynchrony indices between LBBP and LVSP. Although Meiburg *et al.*^19^ described mild to moderate septal delay with LVSP in a virtual pacing model, global PSD did not differ significantly between groups in our cohort, suggesting that regional mechanical delays may not necessarily impact global myocardial mechanics. This observation is also in line with the notion that regional electrical activation patterns (such as lateral wall timing) do not fully capture global electrical synchrony.

To our knowledge, this is the first report describing, in a real-world setting, the similarity in global mechanical performance between LBBP and LVSP during mid- to long-term follow-up, along with LBBAP performance closely matching intrinsic rhythm, paralleling the acute-phase findings by Bertini *et al.*^17^ As previously noted, only the virtual model by Meiburg *et al.*^19^ had predicted potential mechanical differences between LBBAP pacing modalities and a modest advantage of His bundle pacing (HBP) over both, yet without significant impact on global ventricular dynamics, also reported for conventional echocardiographic parameters during mid- to long-term follow-up by Cano *et al.*^20^ Indeed, real-world comparative studies of LBBP or LBBAP vs. HBP have shown that strain and MW indices often fail to detect significant differences between capture types.^29,33^ Furthermore, indirect comparisons between HBP and LBBAP suggest a certain advantage of LBBAP in MW parameters, as highlighted by Prinzen,^31^ with the largest differences consistently observed when contrasting LBBAP with conventional RVP.^34^

Our mechanical findings should also be interpreted in the context of our population, which showed reduced baseline strain values despite preserved ejection fraction. Global longitudinal strain was lower than reference values for healthy individuals, a pattern likely reflecting the advanced age of our cohort, along with a high prevalence of atrial fibrillation, hypertension, and diabetes. While these features are compatible with underlying structural heart disease, the fact that MW parameters remained stable with LBBAP is notable. It suggests that conduction system pacing does not worsen mechanical performance, even in patients with impaired myocardial deformation at baseline. Similarly, Bertini *et al.* also reported preservation of mechanical performance with LBBAP in a cohort where nearly half of the patients had mild-to-moderate systolic dysfunction (LVEF ≥ 40%).^17^ Interestingly, in that study, intrinsic strain values were higher than expected, possibly reflecting patient selection towards more preserved myocardial function. Indeed, in the EMPATHY study, conducted by the same group in a population with baseline characteristics more similar to ours, GLS and MW values were even lower.^29^

Finally, our results may help to elucidate the mechanical effects of LBBAP and its modalities on the left ventricle, but uncertainties remain regarding potential differences between left bundle and fascicular captures,^3^ as well as the performance of the right ventricle,^4,5^ given its electrical activation disparities.

### Limitations

The relatively small sample size represents a limitation of our study. This was mainly related to methodological requirements. Nonetheless, our cohort is consistent in characteristics with previously published series on this topic, and the combined intra-subject and inter-group adjusted analysis allowed for a more comprehensive mechanical characterization. However, unequal group sizes, reflecting the implant strategy (priority of LBBP over LVSP), may have reduced the statistical power for some comparisons between LBBP and LVSP. Also, the potential overlap of electrical criteria used to distinguish between pacing modalities might have led to some degree of misclassification and attenuating mechanical differences between groups, especially since, outside the implant procedure, the observation of transition criteria is rare and difficult to demonstrate during follow-up. Furthermore, differences in heart rate between intrinsic and paced rhythms may also have introduced variability, although the use of MW, less dependent on loading conditions, likely minimized this effect.

Finally, our analysis reflects a single time point in the mid- to long-term post-implantation period and was not powered to evaluate clinical outcomes, like pacing-induced cardiomyopathy. Moreover, our study involved multiple statistical comparisons, and the possibility of chance findings cannot be excluded. Although correction for multiple testing was applied, some results should be interpreted with appropriate caution and viewed as hypothesis generating. Nevertheless, our findings are in line with previously published, provide a mechanical characterization in a chronic setting, and reinforce the need for further studies, ideally integrating advanced echocardiographic tools such as MW with state-of-the-art electrical mapping techniques such as electrocardiographic imaging.^11,35^

## Conclusions

In patients with preserved LVEF and advanced conduction disease, LBBAP maintains global mechanical function close to that seen with intrinsic rhythm at mid- to long-term follow-up while promoting increases in constructive and wasted work. Although MW revealed modest differences between LBBP and LVSP in terms of efficiency, overall mechanical performance was largely similar between modalities. These findings lend support to the preferential use of LBBAP for anti-bradycardia indication when feasible, although further studies with larger, adequately powered cohorts are warranted to confirm them.

## Supplementary Material

euaf271_Supplementary_Data

## Data Availability

The anonymized dataset supporting the findings of this study has been deposited in Zenodo (10.5281/zenodo.17187564). Access to the data is available upon reasonable request to the corresponding author, in line with institutional and ethical regulations.
