# Supplementary material for: Mid- to long-term mechanical performance of left bundle branch area pacing: a comprehensive echocardiographic comparison of capture modalities
Source: Europace. 2025 Oct 29;27(11):euaf271. doi: 10.1093/europace/euaf271 (PMC12619061; doi:10.1093/europace/euaf271)
Supplement: euaf271_Supplementary_Data [file euaf271_supplementary_data.docx]

**SUPPLEMENTARY METHODS.**

**Echocardiography acquisition**

In patients with low percentage of ventricular pacing (<20%), echocardiogram during spontaneous rhythm was first acquired. Then the device was programmed in VVI mode 10 bpm over patient´s intrinsic heart rate and unipolar pacing configuration to ensure LBBA capture without fusion with intrinsic rhythm, and the second echocardiogram was acquired after at least 30 minutes of ventricular pacing.

In the remaining patients (those with >20% ventricular pacing), an echocardiogram during paced rhythm was first acquired. In those with intrinsic rhythm, reprogramming to VVI mode 10 bpm over patient´s intrinsic heart rate and unipolar pacing configuration was performed when necessary. Otherwise, paced echocardiography was acquired with baseline programming, in unipolar configuration. Then, the device was programmed in VVI mode at 30 bpm, and a second echocardiogram was acquired if a stable rhythm of at least 40 bpm appeared (after 30 minutes of waiting).

Baseline programming was set back again in all patients after the acquisition of the echocardiographic images.

**Measurements of electrocardiographic parameters**

At both the implant and follow-up visits, a threshold test in unipolar pacing configuration was performed, and the appearance of QRS transition was sought. The threshold test started at maximum output with a standard pulse width (8 volts @ 0.4 ms) and was conducted in VVI mode, set at least 10 bpm above the patient’s intrinsic heart rate, using unipolar pacing to ensure LBBAP capture without fusion. QRS morphology and RWPT measurements were obtained from the non-selective LBBP capture at a voltage between 2 to 3.5 Volts and standard pulse width of 0.4 ms.

**Measurements of electrical parameters and agreements**

A subset of 40 patients was randomly selected. Interobserver agreement for electrical variables was assessed by comparing the electrocardiographic measurements performed by a first observer with those from the same 40 cases assessed by a blinded second observer. Paced V6-RWPT/V1-RWPT, p-QRS-stim, and p-QRS-onset were measured. Another subset of 20 patients was selected for mechanical synchrony and MW variables. Interobserver agreement for echocardiographic variables was assessed by comparing the echocardiographic measurements performed by a first observer with those from a second observer. PSD, IVMD, GLS, and GWE were measured. Intraclass correlation coefficients (ICCs) were used to calculate the variability between observers. Agreement was considered excellent for ICC >0.74, good for ICC between 0.60 and 0.74, fair for ICC between 0.40 to 0.59, and poor for ICC <0.40. Inter-observer reproducibility was also evaluated with the Bland-Altman analysis (Supplementary Figure S2).

**SUPPLEMENTARY RESULTS**

**Clinical outcomes at follow-up**

Twenty-four patients of the target population with successful LBBAP died (18.4%). Among them, no LBBAP-lead dysfunctions or clinical complications related to the pacemaker were observed at ordinary follow-up before death. Average time between the implant and death was 249.7 ± 176.5 days, and mean age was 87.2 ± 5.9 years old. Five of them (20.8%) were admitted due to heart failure after the implant and before death; in 4 out 5 decompensation was related to previous cardiopathy distinct from rhythm disorder (3 significant valvular diseases and 1 hypertrophic myocardiopathy). Causes of death were as follows: 9 infective diseases; 7 extra-hospitalary cardiac arrest without necropsy study (among which there were 2 severe aortic stenosis); 4 oncologic diseases; 2 decompensated heart failure; 1 stroke; 1 decompensated chronic kidney disease.

**Supplementary Table S1. Baseline characteristics according to presence of spontaneous rhythm.**

|  | Patients with intrinsic rhythm  (n=88) | Patients without intrinsic rhythm (n=50) | P value |
| --- | --- | --- | --- |
| Age (years) | 76.0 (72.2-88.2) | 78.0 (70.7-86.5) | 0.664* |
| Female | 43 (48.9%) | 27 (54%) | 0.859 |
| Hypertension | 79 (89.8%) | 39 (78.0%) | 0.078 |
| Diabetes | 29 (33.0%) | 12 (24.0%) | 0.334 |
| Atrial fibrillation | 37 (42.0%) | 21 (42.0%) | 1.000 |
| Heart Failure | 10 (11.4%) | 5 (10.0%) | 1.000 |
| Coronary heart disease | 11 (12.5%) | 3 (6.0%) | 0.259 |
| COPD | 6 (6.8%) | 3 (6.0%) | 1.000 |
| CKD^†^ | 13 (14.8%) | 6 (12.2%) | 0.799 |
| Medical treatment   - Anticoagulant - Beta-Blocker - ACEI / ARA-II / ARNI - MRA | 41 (47.1%)  25 (28.4%)  58 (65.9%)  8 (9.4%) | 20 (40.8%)  9 (18.4%)  33 (66.0%)  2 (4.2%) | 0.590  0.221  1.000  0.328 |
| Pacing indication   - AVB / BF block / Alternant BBB - Slow AF / SND | 34 (38.6%)  54 (62.4%) | 31 (62.0%)  19 (38.0%) | <0.001 |
| Intrinsic QRS (milliseconds) | 115.3±28.7 | 116.0 (94.5-140.0) | 0.664* |
| Intrinsic wide QRS (≥120 ms) | 28 (44.4%) | 10 (40.0%) | 0.813 |
| Diseased LBB (≥110 ms) | 24 (27.3%) | 14 (28.0%) | 1.000 |
| Pacing modality   - LBBP - LVSP | 63 (71.6%)  25 (28.4%) | 43 (86.0%)  7 (14.0%) | 0.061 |
| Paced QRS (milliseconds)   - From stimulus - From onset | 138.0 (131.0-143.0)  108.0 (101.0-115.7) | 135.0 (130.0-145.7)  107.0 (98.7-114.5) | 0.687*  0.932* |
| Device Type   - SR - DR | 15 (17.0%)  73 (83.0%) | 17 (34.0%)  33 (66.0%) | 0.035 |
| Intrinsic LVEF (%) | 60.0 (58.0-64.2) | 60.0 (60.0-62.7) | 0.794 |
| LVEDD (millimeters) | 44.4±5.5 |  | 0.402 |
| Interventricular septum thickness (millimeters) | 12.0 (11.0-14.0) | 12.0 (10.0-14.0) | 0.125* |
| Left atrial volume (milliliters/m^2^) | 38.0 (31.0-50.0) | 40.0 (34.0-54.5) | 0.262* |
| Ventricular pacing at follow-up (%) | 3.3 (0.1-42.9) | 99.1 (88.0-100.0) | <0.001* |

Values are mean ± standard deviation (SD), median (interquartile range) and n (%).

LBBP: left bundle branch pacing; LVSP: left ventricular septal pacing; COPD: chronic obstructive pulmonary disease; CKD: chronic kidney disease; ACEI: Angiotensin converting enzyme inhibitor; ARA-II: angiotensin II receptor antagonists; ARNI: angiotensin receptor–eprilysin inhibitor; AVB: atrioventricular block; BF block: bifascicular block; BBB: bundle branch block; LVEF: left ventricular ejection fraction; LVEDD: left ventricular end-diastolic diameter.

†Glomerular filtration rate < 60 mL/min/1.73 m2.

*Non-parametric tests.

**Supplementary Table S2. Electrocardiographic characteristics of the LBBAP morphologies at implant and follow-up.**

|  | LBBAP | | | LBBP | | | LVSP | | |
| --- | --- | --- | --- | --- | --- | --- | --- | --- | --- |
|  | Implant | Follow-up | p | Implant | Follow-up | p | Implant | Follow-up | p |
| V1-RWPT (ms) | 118.7±12.0 | 112.3±11.9 | <0.001 | 118.5±12.5 | 111.7±11.8 | <0.001 | 119.3±9.9 | 115.2±12.0 | 0.036 |
| V6-RWPT (ms) | 77.8±11.8 | 74.8±11.9 | 0.001 | 74.4±8.9 | 71.5±10.6 | <0.001 | 89.0±12.6 | 85.6±10.1 | 0.209 |
| Interpeak interval (ms) | 40.6±13.2 | 38.5±12.4 | 0.044 | 44.0±11.8 | 40.6±11.9 | 0.005 | 26.0±8.1 | 29.6±10.7 | 0.039 |
| Paced QRS (from stimulus) | 146.4±14.1 | 139.9±13.9 | <0.001 | 145.8±15.1 | 137.1±12.9 | <0.001 | 147.9±10.9 | 146.8±14.1 | 0.011 |
| Paced QRS (from onset) | 115.7±13.1 | 110.2±13.5 | <0.001 | 115.1±14.1 | 107.7±13.1 | <0.001 | 116.9±10.1 | 116.3±12.6 | 0.001 |

Values are mean ± standard deviation (SD).

LBBAP: left bundle branch area pacing; LBBP: left bundle branch pacing; LVSP: left ventricular septal pacing; RWPT: R wave peak time.

**Supplementary Table S3. 2D-Echocardiographic, strain, and MW indices during LBBAP according to presence of spontaneous rhythm.**

|  | LBBAP | | |
| --- | --- | --- | --- |
|  | **Patients with intrinsic rhythm**  **(n=88)** | **Patients without intrinsic rhythm**  **(n=50)** | **P value** |
| LVEF (%) | 57.0  (55.0-60.0) | 56.5  (55.0-62.0) | 0.908* |
| LVEDV (ml) | 70.0  (55.0-85.0) | 73.5  (55.7-92.5) | 0.509* |
| LVESV (ml) | 31.0  (25.0-40.0) | 33.5  (24.7-45.2) | 0.431* |
| IVMD (ms) | 26.0  (18.0-40.0) | 30.0  (18.0-40-0) | 0.474* |
| PSD (ms) | 59.0  (50.0-70.0) | 60.0  (52.0-73.0) | 0.945* |
| GLS (%) | -15.6±2.5 | -17.2±2.7 | 0.001 |
| GWI (mmHg%) | 1525.8±378.8 | 1707.6±422.9 | 0.013 |
| GCW (mmHg%) | 2147±425.4 | 2236±472.4 | 0.267 |
| GWW (mmHg%) | 249.5  (173.0-363.2) | 231.0  (147.5-287.5) | 0.511* |
| GWE (%) | 88.0  (85.0-92.0) | 89.0  (86.0-92.0) | 0.592* |

Observed values are mean ± standard deviation (SD) or median (interquartile range). LBBAP: left bundle branch area pacing; LVEF: left ventricular ejection fraction; LVEDV: left ventricular end-diastolic volume; LVESV: left ventricular end-systolic volume; IVMD: interventricular mechanical delay; PSD: peak strain dispersion; GLS: global longitudinal strain; GWI: global work index; GCW: global constructive work; GWW: global wasted work; GWE: global work efficiency.

*Non-parametric tests.

**Supplementary Table S4. Adjusted 2D-echocardiographic, strain and MW indices interaction between rhythm and pacing modality.**

|  | F (df) | P value (global) |
| --- | --- | --- |
| LVEF (%) | 0.049 (95.2) | 0.826 |
| LVEDV (ml) | 1.773 (85.0) | 0.187 |
| LVESV (ml) | 6.279 (85.1) | 0.014 |
| IVMD (ms) | 0.273 (95.7) | 0.602 |
| PSD (ms) | 0.174 (105.2) | 0.677 |
| GLS (%) | 0.014 (78.9) | 0.905 |
| GWI (mmHg%) | 0.041 (85.5) | 0.840 |
| GCW (mmHg%) | 1.017 (78.8) | 0.316 |
| GWW (mmHg%) | 0.220 (84.8) | 0.640 |
| GWE (%) | 0.745 (91.1) | 0.390 |

df: degrees of freedom; LVEF: left ventricular ejection fraction; LVEDV: left ventricular end-diastolic volume; LVESV: left ventricular end-systolic volume; IVMD: interventricular mechanical delay; PSD: peak strain dispersion; GLS: global longitudinal strain; GWI: global work index; GCW: global constructive work; GWW: global wasted work; GWE: global work efficiency.

**Supplementary Table S5. Unadjusted comparison of deltas between LBBP and LVSP.**

| Change from paced to intrinsic beats  (paced-intrinsic=Δ) | LBBP  (n=63) | LVSP  (n=25) | P value |
| --- | --- | --- | --- |
| ΔLVEF (%) | -0.8±4.1 | -0.3±4.2 | 0.560 |
| ΔIVMD (ms) | -4.2±14.9 | -1.0±19.5 | 0.423 |
| ΔPSD (ms) | -7.0 (-24.0 / +8.0) | -2.5 (-16.2 / +5.2) | 0.857* |
| ΔGLS (%) | +0.4 (-0.7 / +2.6) | -0.1 (-1.0 / +1.7) | 0.800* |
| ΔGWI (mmHg%) | -1.9±409.2 | +95.9±431.6 | 0.354 |
| ΔGCW (mmHg%) | +81.0 (-136.0 / +266.0) | +269.5 (-11.2 / +602.0) | 0.182* |
| ΔGWW (mmHg%) | +43.5±135.1 | +63.8±101.1 | 0.527 |
| ΔGWE (%) | -0.6±4.9 | -1.6±3.9 | 0.418 |

Values are mean ± standard deviation (SD) or median (interquartile range). LBBP: left bundle branch pacing; LVSP: left ventricular septal pacing; LVEF: left ventricular ejection fraction; IVMD: interventricular mechanical delay; PSD: peak strain dispersion; GLS: global longitudinal strain; GWI: global work index; GCW: global constructive work; GWW: global wasted work; GWE: global work efficiency.

*Non-parametric tests

**Supplementary Table S6. Interobserver agreement of the electrocardiographic and echocardiographic parameters.**

| Parameter | Intraclass correlation coefficient | P value |
| --- | --- | --- |
| V6-RWPT (ms) | 0.967 | <0.001 |
| V1-RWPT (ms) | 0.983 | <0.001 |
| P-QRS-onset (ms) | 0.925 | <0.001 |
| P-QRS-stim (ms) | 0.901 | <0.001 |
| PSD | 0.878 | 0.015 |
| IVMD | 0.833 | 0.031 |
| GLS | 0.870 | 0.013 |
| GWE | 0.812 | 0.039 |

IVMD: interventricular mechanical delay**;** GLS: global longitudinal strain; GWE: global work efficiency; p-QRS-onset: paced QRS duration measured from onset; P**-**QRS-stim: paced QRS duration measured from pacing spike; PSD: peak strain dispersión; RWPT: R wave peak time.

**Supplementary Figure S1. Electrocardiographic changes between implant and follow-up.**


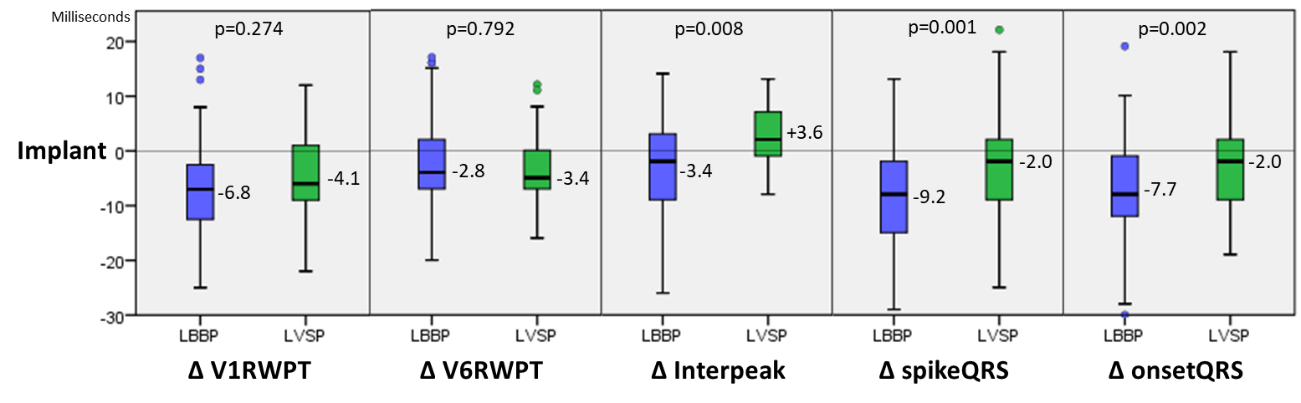


Box-plot graphics showing the change between implant and follow up in V1-RWPT, V6-RWPT, Interpeak interval, paced QRS from spike, and paced QRS from onset, among LBBP and LVSP captures. Mean values are superimposed. LBBP: left bundle branch pacing; LVSP: left ventricular septal pacing; RWPT: R-wave peak time.

**Supplementary Figure S2. Bland-Altman analysis for inter-observer agreement in GWE, GLS, PSD and IVMD.**


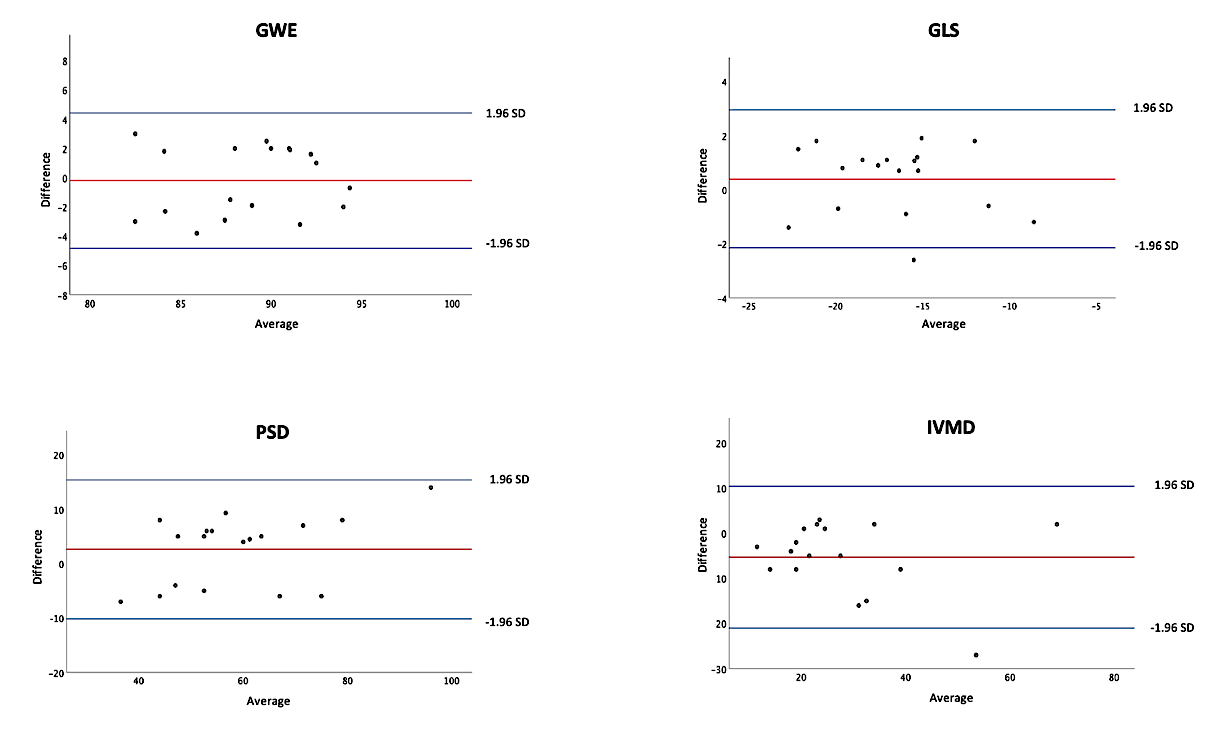


The x-axis shows the mean of the two observers; the y-axis shows their difference (Observer 1 − Observer 2). The central solid line is the mean bias; the upper and lower lines are the 95% limits of agreement (bias ±1.96 SD).

IVMD: interventricular mechanical delay**;** GLS: global longitudinal strain; GWE: global work efficiency; PSD: peak strain dispersion; SD: standard deviation.
